# A Review of the Impact of Sjögren’s Syndrome and/or the Presence of Anti-Ro/SS-A Antibodies on Therapeutic Strategies for Rheumatoid Arthritis

**DOI:** 10.3390/jcm14020568

**Published:** 2025-01-17

**Authors:** Yoshiro Horai, Shota Kurushima, Toshimasa Shimizu, Hideki Nakamura, Atsushi Kawakami

**Affiliations:** 1 Department of Rheumatology, Sasebo City General Hospital, Sasebo 857-8511, Japan; ssbkrshm@gmail.com; 2 Department of Immunology and Rheumatology, Division of Advanced Preventive Medical Sciences, Nagasaki University Graduate School of Biomedical Sciences, Nagasaki 852-8523, Japan; toshimasashimizu2000@yahoo.co.jp (T.S.); atsushik@nagasaki-u.ac.jp (A.K.); 3 Clinical Research Center, Nagasaki University Hospital, Nagasaki 852-8501, Japan; 4 Division of Hematology and Rheumatology, Department of Medicine, Nihon University School of Medicine, Tokyo 173-8610, Japan; nakamura.hideki@nihon-u.ac.jp

**Keywords:** anti-Ro/SS-A antibodies, disease-modifying anti-rheumatic drugs, lymphoproliferative disorders, rheumatoid arthritis, Sjögren’s syndrome

## Abstract

Rheumatoid arthritis (RA) is an immune-mediated disease characterized by polyarthritis that affects the small joints of the bilateral upper and lower extremities. RA shares several common clinical symptoms with Sjögren’s syndrome (SS), another rheumatic disease caused by the lymphocytic infiltration of exocrine glands, with dry eye and dry mouth being the two most common symptoms. Anti-Ro/SS-A antibodies, a diagnostic biomarker of SS, are positive in patients with RA at a certain rate. The coexistence of SS and/or positivity for anti-Ro/SS-A antibodies in patients with RA influences disease activity and the effectiveness of several classes of disease-modifying antirheumatic drugs (DMARDs). Furthermore, RA, SS, and certain DMARDs, including methotrexate, are associated with the onset of lymphoproliferative disorders (LPD). In contrast, several biological DMARDs, such as tocilizumab and rituximab, are plausible options without the risk of LPD relapse. Considering the results of the studies introduced in this article, RA with SS and/or positivity for anti-Ro/SS-A antibodies could be considered a phenotype different from isolated RA from the perspective of refractoriness to DMARD therapy and LPD risk. Hence, rheumatologists should observe caution when choosing DMARDs. Further studies are needed to establish the appropriate treatment for patients with RA, SS, and/or the presence of anti-Ro/SS-A antibodies.

## 1. Introduction

Rheumatoid arthritis (RA) is an immune-mediated disease characterized by polyarthritis that typically affects the small joints of the bilateral upper and lower extremities, and genetic and environmental factors are implicated in this condition [1,2]. The European Alliance of Associations for Rheumatology (EULAR, formerly European League Against Rheumatism) recommends introducing disease-modifying antirheumatic drugs (DMARDs) immediately after RA diagnosis [3]. Of the various DMARDs, methotrexate (MTX), a conventional synthetic DMARD (csDMARD), is currently considered the first-line therapeutic option for RA [3]. However, MTX-associated lymphoproliferative disorder (MTX-LPD), another form of iatrogenic immunodeficiency-associated LPD (OIIA-LPD), is a severe adverse effect of MTX [4,5]. Other csDMARDs, such as tacrolimus (TAC) and iguratimod (IGU), as well as biological DMARDs (bDMARDs) such as tumor necrosis factor inhibitors (TNFi), have been associated with the development of LPD [5,6,7]. In many cases, MTX-LPD can be expected to achieve spontaneous regression (SR) after the termination of MTX [8]. However, a certain number of cases of LPD associated with DMARDs, including MTX, may progress and cause hemophagocytic syndrome, even after achieving partial remission with the cessation of DMARDs [9]. RA, especially with high disease activity, is presumed to contribute to LPD development [10,11]; however, rheumatologists are required to cautiously choose DMARDs owing to their adverse effects, including LPD.

Sjögren’s syndrome (SS), also known as Sjögren’s disease (SD) [12], is another rheumatic disease caused by the lymphocytic infiltration of exocrine glands, with dry eye and dry mouth being the two most common symptoms [13,14,15,16,17,18]. It is divided into primary SS (pSS) and secondary SS (sSS), depending on the presence or absence of other rheumatic diseases [19]. Anti-Ro/SS-A antibodies and anti-La/SS-B antibodies are diagnostic biomarkers for SS. In contrast, the isolated positivity of anti-La/SS-B antibodies has not been reported as useful in clinical practice [20]. Currently, anti-Ro/SS-A antibodies, but not anti-La/SS-B antibodies, are included in the 2016 American College of Rheumatology/European League Against Rheumatism classification criteria for pSS [21,22]. The positive rate of anti-Ro/SS-A antibodies was estimated to be 72% [23]. When patients with SS are diagnosed with RA, they are considered to have sSS, irrespective of whether SS precedes RA [24]. pSS can involve extraglandular manifestations, including articular symptoms; reportedly, the symmetrical form was more common compared to monoarthritis in 16% of pSS-manifested arthritis [23]. A study reported that 22.1% of patients with pSS were positive for anti-cyclic citrullinated peptide antibodies, a diagnostic biomarker for RA [25]. In contrast, RA with sSS tends to have higher disease activity compared to RA without sSS [26]. Reportedly, 3–15% of patients with RA were positive for anti-Ro/SS-A antibodies, which might be positive in rheumatic diseases other than SS and RA, such as systemic lupus erythematosus [27], dermatomyositis (DM) [28], and systemic sclerosis [29]. The presence of anti-Ro/SS-A antibodies was associated with a decrease in therapeutic effects of several classes of DMARDs; that is, patients with RA that were positive for anti-Ro/SS-A antibodies and negative for anti-Ro/SS-A antibodies might represent distinct clinical subsets and may require different therapeutic strategies [27,30]. Furthermore, sicca symptoms may develop as extra-articular manifestations of RA [1,31]. When physicians encounter patients with sicca symptoms complicating arthritis, differential diagnoses of RA, pSS, and RA with sSS are required, and the existence of sSS or positive anti-Ro/SS-A antibodies should be considered when choosing DMARDs. The therapeutic strategy used to manage arthritis in pSS is not the same but shares features with those used for RA. Notably, several DMARDs, such as MTX, hydroxychloroquine (HCQ), and rituximab (RTX), are considered for the treatment of arthritis in patients with pSS [32]. However, in addition to DMARDs being potentially associated with OIIA-LPD, the existence of SS, as well as RA, is thought to be a risk factor for the development of LPD [10,33], suggesting the need to consider OIIA-LPD when using DMARDs as a therapy against arthritis in pSS. In a study of patients with RA treated with tofacitinib, a Janus kinase inhibitor (JAKi), a larger percentage of patients had sSS in the lymphoma group compared to the control group [34]. The relative risk of LPD in patients with RA and sSS compared to those without sSS is unclear; however, these findings and the above-mentioned OIIA-LPDs suggest that rheumatologists should pay attention to LPD when choosing DMARDs, especially in patients with RA and sSS. The association between anti-Ro/SS-A antibody positivity without a clinical diagnosis of SS and LPD has yet to be established. However, there is a report suggesting that no lymphoma cases were observed in patients with pSS negative for anti-Ro/SS-A antibodies and anti-La/SS-B antibodies [35]. Considering this, along with the presumed link between high RA disease activity and LPD [11] and the refractoriness to various DMARDs associated with anti-Ro/SS-A antibodies [27,30], the presence of anti-Ro/SS-A antibodies—even without a clinical diagnosis of SS—might be another important factor for rheumatologists to consider when choosing DMARDs. This is to ensure efficacy and safety when dealing with such cases, in contrast to patients negative for anti-Ro/SS-A antibodies.

In this review, we discuss previous findings and suitable treatments for patients with RA coexisting with SS or positive anti-Ro/SS-A antibodies. We focus on their efficacy and effectiveness in managing arthritis and sicca symptoms and the risk of LPD. We aim to contribute to the better understanding of and appropriate treatment for patients with RA and sSS and/or presence of anti-Ro/SS-A antibodies.

## 2. Influence of the Coexistence of sSS or Positiveness for Anti-Ro/SS-A Antibodies on the Disease Activity of RA and the Relationship Between RA, SS, Anti-Ro/SS-A Antibodies, and LPD

### 2.1. Complications of SS in Patients with RA and Its Influence on Disease Activity

Previously reported complication rates of sSS in patients with RA were 6.3–7.3%, varying depending on the applied SS classification criteria, such as the 1993 European Classification criteria and the 2002 American–European Consensus Group classification criteria for SS [36]. Complications of sSS are factors that predict high disease activity and the refractoriness of RA. Laroche et al. conducted a retrospective study of erosive RA, comparing patients with and without sSS, and the results showed a higher disease activity score (DAS) and a higher number of prescribed DMARDs in patients with sSS compared to those without sSS [26]. Higher disease activity of RA with sSS compared to RA without sSS was also confirmed in a meta-analysis, which consisted of 16 observational study papers conducted by Tomizawa et al. [37]. Alp et al. analyzed 302 cases of RA to clarify the characteristics of difficult-to-treat RA (D2T RA) and clarified that the D2T RA group had higher disease activity assessed using the DAS28-erythrocyte sedimentation rate (ESR), and a statistically significant higher rate of sSS was found in the D2T RA group than in the non-D2T RA group [38]. Data from the Swiss Clinical Quality Management (SCQM) registry revealed a higher DAS, power Doppler ultrasound score, and more severe bone erosion in patients with RA and sSS than in those without sSS [39]. The decreased effectiveness of TNFis and MTX associated with the positivity of anti-Ro/SS-A antibodies has been reported in a population of patients with RA and without sSS; that is, anti-Ro/SS-A antibody positivity might also be a refractory factor of RA [27,30].

### 2.2. The Incidence of LPD in RA

RA is a risk factor for LPD development. Smedby et al. reported that the odds ratio of developing non-Hodgkin lymphoma in patients with RA compared to the control subjects was 1.5 with a 95% confidence interval of 1.1–1.9, and the subanalyses revealed that the use of immunosuppressive drugs such as MTX was significantly associated with the development of non-Hodgkin lymphoma [10]. The Epstein–Barr virus (EBV), involved in the pathophysiology of RA [1] and SS [40], is associated with the development of OIIA-LPD, and the rate of EBV positivity was 57% among patients with MTX-associated diffuse large B cell lymphoma [4]. The frequency of LPD in patients with RA is presumed to be higher in Japan than in Western countries; a standardized incidence rate of 3–6 was estimated in Japanese patients with RA [41]. In a multicenter retrospective study of 216 cases of MTX-LPD conducted in Japan, two-thirds of the study participants achieved SR after termination of MTX, ~74% of whom achieved complete or partial regression at 2 weeks after the termination of MTX, whereas the remaining one-third of the patients did not [8].

### 2.3. LPD in Patients with RA and sSS and/or the Presence of Anti-Ro/SS-A Antibodies

The above-mentioned study population included 35 (16.6%) patients with sSS, and no significant difference was observed between the SR group and the non-SR group in terms of the complication rate of sSS. Regarding pathological diagnosis, EBV-positive mucocutaneous ulcers were more frequently observed in the SR group than in the non-SR group [8]. Dawson et al. investigated the expression of EBV in lymphoma tissues obtained from patients with RA or pSS using in situ hybridization. In addition, 5/19 cases of RA and 1/6 cases of pSS were positive for EBV; 4/5 cases of RA positive for EBV were taking MTX; and the remaining patients had a history of MTX use before the lymphoma diagnosis [42]. These findings suggest that EBV may be a common pathogenic factor for both MTX-LPD in patients with RA and LPD in patients with SS. Whether the presence of anti-Ro/SS-A antibodies, irrespective of SS, is linked to the future development of LPD remains unclear; however, anti-Ro/SS-A antibodies might be considered as a risk factor for lymphoma in clinical practice. Bodakçi recently reported that no patients developed lymphoma in a study of 184 cases of pSS negative for anti-Ro/SS-A antibodies and anti-La/SS-B antibodies [35]. This suggests anti-Ro/SS-A antibodies may be involved in the development of lymphoma.

## 3. Choice of DMARDs Considering Efficacy, Effectiveness of Refractoriness, and Risk of OIIA-LPD

### 3.1. csDMARDs

#### 3.1.1. Methotrexate

Even with the introduction of molecular targeted therapies for RA, such as bDMARDs and JAKis, MTX is considered the mainstay treatment for RA and can be used as a monotherapy or in combination with other csDMARDs and/or bDMARDs. In this context, MTX is often referred to as the “anchor drug” in the treatment of RA [43]. The use of synthetic immunosuppressive agents, including MTX, as a glucocorticoid-sparing agent was included in the EULAR recommendations for SS published in 2019 [44]. In 2024, the British Society for Rheumatology guidelines for the management of SD introduced a drug of choice for arthritis in SD [12]. By contrast, the presence of anti-Ro/SS-A antibodies has been suggested as a refractory factor to MTX in RA. Waki et al. compared the characteristics of patients with RA positive for anti-Ro/SS-A antibodies with those negative for anti-Ro/SS-A antibodies. They found a statistically significantly lower achievement rate of low disease activity determined by the DAS28 C-reactive protein in patients with RA positive for anti-Ro/SS-A antibodies, when compared to those negative for anti-Ro/SS-A antibodies, 6 months after MTX treatment [30].

MTX-LPDs are the most well-known form of OIIA-LPDs used for treating RA. SR after MTX withdrawal can be expected in a certain number of patients [4,5]. Hence, MTX should be terminated at the time of diagnosis.

#### 3.1.2. Leflunomide

In the EULAR recommendations for managing RA, leflunomide (LEF) is included in Phase I of the algorithm as an option in cases where MTX is contraindicated [3]. However, improvements in sicca symptoms due to LEF, measured by Schirmer’s test and sialometry in pSS, were not considered statistically significant in a phase II open-label pilot study [45]. Shahin et al. analyzed the effects of LEF as an additional therapy for MTX in 45 patients with RA and sSS and 30 patients with RA. An improvement in DAS28-ESR for RA was found in both groups; however, deterioration of eye dryness measured by the modified Schirmer’s I test was found only in the RA and sSS groups, and this might be attributable to the inhibitory effects of LEF on nitric oxide, which is assumed to maintain the homeostasis of the ocular surface [46]. Considering these reports, rheumatologists should be cautious about the adverse effects on the eyes when considering LEF in patients with RA complicated by SS. LEF-associated LPD has been reported in case reports; Fujioka et al. reported a case of pulmonary diffuse large B-cell lymphoma after 40 years of LEF administration [47], and Benzerdjeb et al. reported a case of cardiac B-cell lymphoma after 10 years of LEF and 2 years of infliximab (IFX) administration [48].

#### 3.1.3. Hydroxychloroquine

In the EULAR recommendations for RA, HCQ, due to its limited effect on preventing joint destruction, is positioned as an alternative DMARD for early and mild RA in which the administration of other DMARDs, such as MTX, is not appropriate [3]. Demarchi et al. retrospectively analyzed the effectiveness of HCQ on the extraglandular manifestations of pSS and found lower complication rates of extraglandular manifestations, including arthritis, in patients treated with HCQ compared to those treated without HCQ [49]. The influence of HCQ on LPD in patients with RA is unclear; however, Zhang et al. found that in a retrospective study of patients with systemic lupus erythematosus—another rheumatic disease positive for anti-Ro/SS-A antibodies at a certain rate—fewer patients with hematological malignancies, including lymphoma, took HCQ compared to those without hematological malignancies [50]. The results suggest a protective effect of HCQ against hematological malignancies, including LPD.

#### 3.1.4. Tacrolimus

TAC is a calcineurin inhibitor that has been approved as an antirheumatic drug in several countries, including Japan [51]. Xu et al. reported that TAC improved refractory immune thrombocytopenia associated with pSS, which was attributed to its reductive effects on Th1 cytokine expression [52]; however, the effects of TAC on RA with sSS or RA positive for anti-Ro/SS-A antibodies are yet to be analyzed. TAC has been reported as a risk factor for MTX-LPD in a bDMARD-naïve population, suggesting the possibility of an additive effect of MTX and TAC on LPD development [5].

#### 3.1.5. Iguratimod

Nagata et al. reported a case of RA that developed MTX-LPD, which regressed after the termination of MTX; however, relapse was found after 4 months of IGU use, which might be attributable to the suppressive effects on T helper 17 cell differentiation in IGU [6]. Hagihara et al. reported a case of MTX-associated intravascular large B-cell lymphoma that regressed after the termination of MTX, but relapse was observed soon after the introduction of IGU [53]. Whether the two cases were complicated with sSS or were positive for anti-Ro/SS-A antibodies is unknown; however, caution about OIIA-LPD might be necessary even when IGU is used, especially after the regression of MTX-LPD.

### 3.2. Biological Disease-Modifying Antirheumatic Drugs (bDMARDs)

#### 3.2.1. Tumor Necrotic Factor Inhibitors

The findings of a prospective study of patients with RA treated with IFX or etanercept (ETN), conducted by Cavazzana et al., showed comparable effectiveness measured by DAS between patients positive for anti-Ro/SS-A antibodies and negative for anti-Ro/SS-A antibodies. However, the number of patients positive for anti-Ro/SS-A antibodies was limited to 17, and subanalyses distinguishing IFX from ETN were not found in the paper [54]. In a retrospective study of patients with erosive RA conducted by Laroche et al., more types of TNFis were prescribed; however, the effective rate was lower in 39 patients with sSS compared to 39 patients without sSS, suggesting the limited effectiveness of TNFis on RA with sSS [26]. An analysis based on 337 patients with sSS and 5974 patients without sSS from the above-mentioned SCQM registry revealed a lower continuation rate of TNFis in patients with RA and sSS, and the investigators suggested the need for the early consideration of treatment modalities other than TNFis [39]. The emergence of anti-drug antibodies is an important factor contributing to treatment failure and adverse effects in biological drug therapies. Notably, the rate for TNFis has been reported to be relatively higher compared to other bDMARDs [55]. Of the several types of TNFis, IFX has been reported to show decreased efficacy in patients with RA who are positive for anti-Ro/SS-A antibodies [27].

The use of TNFis in inflammatory diseases, including RA, is associated with an increased risk of lymphoma [7]. In contrast, several other studies have reported non-significant results on the relationship between TNFis and LPD [56,57]. To the best of our knowledge, there are currently no reports on whether sSS or anti-Ro/SS-A antibodies influence the pharmacological dynamics, therapeutic effects, or development of LPD in patients with RA treated with TNFis other than IFX, ETN, and adalimumab (ADA).

##### 3.2.1.1. Infliximab

The efficacy of IFX on the disease activity of pSS measured using the visual analog scale and tear/saliva secretion tests was denied in a randomized controlled trial [58]. Matsudaira et al. reported that patients with RA positive for anti-Ro/SS-A antibodies showed a significantly lower therapeutic response and a higher discontinuation rate due to the inefficacy of anti-TNF therapies, especially IFX, when compared to patients with RA who were negative for anti-Ro/SS-A antibodies [27]. Hagiwara et al. analyzed 110 patients with RA treated with bDMARDs, including 59 patients treated with IFX, and analyzed the effectiveness of anti-Ro/SS-A antibodies. In patients treated with IFX, DAS28-ESR improved significantly in patients negative for anti-Ro/SS-A antibodies but not in patients positive for anti-Ro/SS-A antibodies, and this was attributable to the low serum transforming growth factor-β1 levels, the human antichimeric antibody and seroconversion rate of antinuclear antibody in patients positive for anti-Ro/SS-A antibodies [59]. In a systematic review of anti-drug antibodies against biological drugs by Strand et al., IFX and IFX biosimilars had the highest immunogenicity rates among the drugs analyzed, and this could be attributed to the structural characteristics of these chimeric TNFis [55].

##### 3.2.1.2. Adalimumab

In the above-mentioned systematic review of anti-drug antibodies against biological drugs reported by Strand et al., ADA had the highest immunogenicity rate, followed by IFX and IFX biosimilars [55]. Chen et al. investigated patients with RA treated with ADA in combination with MTX and compared the characteristics of patients positive for antibodies against ADA to those of patients negative for antibodies against ADA. They found that anti-TROVE2 antibodies, a class of anti-Ro/SS-A antibodies against Ro60, were preferentially distributed in the patients positive for antibodies against ADA, and the positivity of anti-TROVE2 antibodies was identified as an independent factor for developing antibodies against ADA. Furthermore, at 24 weeks, a significantly lower ADA concentration was found among the patients positive for antibodies against ADA than among those negative for antibodies against ADA. The titers of antibodies against ADA were inversely correlated with plasma ADA concentrations, and there was a significantly lower rate of low disease activity at 24 weeks in the patient group positive for antibodies against ADA compared to the group negative for antibodies against ADA. These findings indicate a decreased effectiveness similar to that of IFX due to anti-drug antibodies in patients with RA who are positive for anti-Ro/SS-A antibodies [60].

##### 3.2.1.3. Etanercept

The rate of emergence of anti-drug antibodies against ETN was lower than that against other TNFis, such as IFX and ADA [55]. Considering this and the above-mentioned report of combined data on IFX and ETN by Cavazzana et al. [54] as well as that on IFX by Matsudaira et al. [27], the extent of the attenuation of effectiveness related to anti-Ro/SS-A antibodies of ETN seemed less than that of IFX. However, in the above-mentioned study from the SCQM registry, the continuation rate of ETN in patients with RA and sSS was comparable to that in other classes of TNFis [39].

Table 1 summarizes the efficacy and risks of TNFis based on anti-Ro/SS-A antibody status.

#### 3.2.2. Tocilizumab

In the above-mentioned study from the SCQM registry, bDMARDs other than TNFis, including interleukin-6 (IL-6) receptor inhibitors, were suggested as options for patients with RA and sSS based on their better continuation rates compared to TNFi [39]. In a study by Hagiwara et al., a decreased effectiveness associated with positive anti-Ro/SS-A antibodies in patients treated with IFX was not observed in patients treated with tocilizumab (TCZ) [59].

TCZ was also presumed to be a suitable option from the perspective of the risk or history of LPD in patients with RA. In a multicenter randomized prospective study conducted in Japan, the efficacy of TCZ monotherapy measured by DAS28 at 52 weeks was comparable to that of TCZ in combination with MTX. However, the preventive effects on radiographic progression measured by the modified total Sharp score were different from those in combination with MTX [61]. IL-6 signaling is presumed to be associated with the development of lymphoma, and it has been suggested that the blockade of IL-6 induced by TCZ might reduce lymphoma development, even among patients with RA taking MTX [5]. Recently, TCZ was reported to be suitable for treating patients with RA after LPD development based on the low frequency of LPD relapse associated with TCZ found in a nationwide study conducted in Japan [62]. Considering the effectiveness of TCZ—even as monotherapy without MTX, regardless of anti-Ro/SS-A antibody status, along with its lower association with the onset of LPD—it appears to be a promising option for treating patients with RA and sSS and/or positive for anti-Ro/SS-A antibodies. This recommendation is made with the understanding of the condition that risks such as bacterial infection, which tend to be diagnosed later due to TCZ’z pharmacological characteristic of blocking the IL-6 receptor, result in the low or no elevation of C-reactive protein levels [63].

#### 3.2.3. Abatacept

Abatacept (ABT), a cytotoxic T-lymphocyte antigen-4 fusion protein that modulates the co-stimulatory signal for T-cell activation has been explored for its efficacy in autoimmune diseases, including pSS, in addition to RA. Adler et al. reported that patients with pSS treated with ABT showed a significant increase in salivary production [64]. By contrast, in the ASAP study, ABT treatment improved the EULAR SS Disease Activity Index and EULAR SS Patient Reported Index but not the tear and saliva secretion test results in patients with pSS [65]. The effectiveness of ABT in patients with RA and sSS was investigated in a multicenter prospective study in Japan. In the Rheumatoid Arthritis with Orencia Trial toward Sjögren’s syndrome Endocrinopathy (ROSE) trial, statistically significant improvements in the disease activity of RA were measured using a simplified disease activity index. In tear secretion was measured using Schirmer’s test. Additionally, improvement in saliva secretion measured using Saxon’s test was observed in patients with Greenspan grade 1 or 2 in labial salivary gland biopsy [66,67]. The effectiveness of ABT in RA and sSS was also confirmed in a study with a larger number of patients (the ROSE II trial) [68]. Considering these findings, ABT is expected to exert effects on arthritis and salivary gland inflammation in patients with RA and sSS. In a study from the SCQM registry, ABT and TCZ were therapeutic options with better continuation rates than TNFi in patients with RA and sSS [39].

For RA and anti-Ro/SS-A antibodies, in the study by Hagiwara et al., ABT was effective for patients with RA who were positive for anti-Ro/SS-A antibodies when compared to patients with RA who were negative for anti-Ro/SS-A antibodies [59]. However, another study assessing the influence of anti-Ro/SS-A antibodies on the effectiveness of RA treatment with a larger patient number reported by Endo et al. demonstrated a lower effectiveness when measured using DAS28-ESR and DAS28-CRP, as well as through musculoskeletal ultrasound findings of synovitis [69]. Regarding LPD, an increased risk of lymphoma associated with ABT was not found in three observational data sources from Europe or North America, but it was found in one Swedish registry of RA. These findings highlighted the need for further studies with larger patient numbers [70].

#### 3.2.4. Rituximab

RTX was included in the bDMARDs group, and its continuation was better in patients with RA and sSS than that of TNFis in the SCQM registry [39]. In a retrospective study of patients with erosive RA conducted by Laroche et al., RTX was administered more often in patients with sSS, diagnosed based on salivary gland biopsy findings, positivity of anti-Ro/SS-A antibodies, or both, compared to patients without sSS. A better efficacy rate was confirmed in patients with sSS than in those without [26].

RTX therapy, such as in rituximab-cyclophosphamide, doxorubicin, vincristine, and prednisolone, is part of the chemotherapy used for MTX-LPD [4]. In 2021, the American College of Rheumatology Guideline for the Treatment of RA mentioned RTX as a conditionally recommended option for patients with a history of LPDs and moderate-to-high disease activity of RA. This was based on the presumption that RTX does not increase the recurrence or exacerbation of LPDs [71]. Considering SS as a potential risk factor for LPD and given the report by Laroche, Degboe, and Constantin [26], RTX is a promising option for patients with RA and sSS despite the inherent risks of RTX, such as opportunistic infections.

### 3.3. Janus Kinase Inhibitors

Similarly to ABT, the efficacy of JAKis has been explored in autoimmune diseases other than RA, including SS [72]. However, the number of studies on the effectiveness of JAKis on SS or the positivity of anti-Ro/SS-A antibodies is relatively limited compared to other classes of DMARDs. In the aforementioned SCQM registry, the continuation rate of JAKis was superior to that of TNFis [39]. Matsumoto et al. reported a case of RA positive for anti-Ro/SS-A antibodies complicated by nail patella syndrome in which combination therapy with MTX and biological drugs was not satisfactorily effective but replacement with JAKi was effective. Details such as the types of bDMARDs and JAKis are not provided in the paper; however, this case might illustrate the merits of JAKis on RA positivity for anti-Ro/SS-A antibodies refractory to other DMARDs [73]. The efficacy of filgotinib in a group consisting of patients with pSS and sSS accompanied by other autoimmune diseases, such as RA or systemic lupus erythematosus, DM, and positive anti-Ro/SS-A or anti-La/SS-B antibodies, was explored in a randomized, double-blind, placebo-controlled study. However, the result was not statistically significant [74].

Failure to prove the non-inferiority of JAKis to TNFis in the occurrence rates of major adverse cardiovascular events and cancer in ORAL Surveillance [75] was thought to influence physicians’ decision to choose molecular-targeted drugs for patients with RA [76], whereas no control group was set in ORAL Surveillance [77]. From the perspective of MTX-LPD, the efficacy of several types of JAKis as monotherapy has been ascertained in clinical studies; however, the effects of JAKis would be maximized when used in combination with MTX [72]. As mentioned above, in a study of tofacitinib, a larger percentage of patients had sSS in a group of patients with RA and lymphoma compared to those without lymphoma. However, the concomitant usage rate and weekly dose administration of MTX were also higher in the lymphoma group [34]. There is also the possibility that sSS itself might affect the occurrence rate of lymphoma.

**Figure 1:**
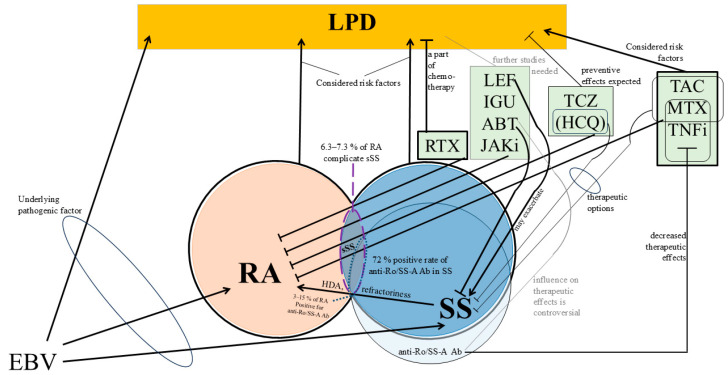
Presumed association between RA, sSS, anti-Ro/SS-A antibodies; therapeutic effects of DMARDs; and LPD discussed in this article.

The number of studies on RA with sSS and/or anti-Ro/SS-A antibodies is limited. Hence, further investigations are required. The use of bibliometric analyses and scientific mapping methods may enhance our understanding of RA with sSS and/or anti-Ro/SS-A antibodies [78]. Furthermore, future advancements in nanotechnology and medicine might lead to the development of specialized therapies for patients with RA and sSS and/or anti-Ro/SS-A antibodies [79]. Figure 1 summarizes the presumed associations between RA, sSS, anti-Ro/SS-A antibodies, and the therapeutic effects of DMARDs and LPD discussed in this article.

## 4. Conclusions

Based on the accumulated evidence presented in this study, RA with sSS and/or positivity for anti-Ro/SS-A antibodies could be considered a phenotype different from isolated RA, requiring different therapeutic strategies. Rheumatologists should consider the possibility that the effects of several classes of DMARDs may be decreased in the presence of sSS and/or positivity for anti-Ro/SS-A antibodies. RA is a risk factor for LPD; and SS associated with EBV and anti-Ro/SS-A antibodies might play a role in facilitating LPD, suggesting the need for caution when administering DMARDs, which are believed to be associated with LPD development in patients with RA and sSS and/or positivity for anti-Ro/SS-A antibodies.

## Figures and Tables

**Table 1:** The efficacy and risks of TNFis based on anti-Ro/SS-A antibody status discussed in this article.

TNFis	Efficacy	Risks
IFX	Significantly lower therapeutic response in patients with anti-Ro/SS-A antibodies	Higher discontinuation rate
ADA	Decreased effectiveness due to ADA in patients with anti-Ro/SS-A antibodies	Developing anti-drug antibodies
ETN	Might be comparable either in patients with anti-Ro/SS-A antibodies or patients without anti-Ro/SS-A antibodies (evidence is limited)	Developing anti-drug antibodies (would be lower than IFX and ADA)

## Data Availability

Not applicable.

## Abbreviations

Ab	Antibodies
ABT	Abatacept
DMARDS	Disease modifying antirheumatic drugs
EBV	Epstein–Barr virus
EULAR	European Alliance of Associations for Rheumatology
HCQ	Hydroxychloroquine
HDA	High disease activity
IGU	Iguratimod
JAKi	Janus kinase inhibitor
LEF	Leflunomide
LPD	Lymphoproliferative disorder
MTX	Methotrexate
RA	Rheumatoid arthritis
RTX	Rituximab
ROSE	Rheumatoid arthritis with Orencia trial toward Sjögren’s syndrome Endocrinopathy
SS	Sjögren’s syndrome
sSS	Secondary SS
TAC	Tacrolimus
TCZ	Tocilizumab
TNFi	Tumor necrosis factor inhibitor
